# Urban malaria and population mobility in sub-Saharan Africa: systematic review and meta-analysis

**DOI:** 10.1186/s12936-025-05508-1

**Published:** 2025-08-18

**Authors:** Hailu Merga, Teshome Degefa, Zewdie Birhanu, Ming-Chieh Lee, Guiyun Yan, Delenasaw Yewhalaw

**Affiliations:** 1 Department of Epidemiology, Institute of Health, Jimma University, Jimma, Ethiopia; 2 School of Medical Laboratory Sciences, Institute of Health, Jimma University, Jimma, Ethiopia; 3 Tropical and Infectious Diseases Research Centre, Jimma University, Jimma, Ethiopia; 4 Departement of Health, Behavior, and Society, Faculty of Public Health, Jimma University, Jimma, Ethiopia; 5 Program in Public Health, University of California at Irvine, Irvine, USA

**Keywords:** Urban malaria, Prevalence, Epidemiology, Determinants, Systematic review, Meta-analysis, Sub-Saharan Africa

## Abstract

**Background:**

Malaria control in African cities faces challenges mainly due to rapid and unplanned urbanization and the spread of the new urban malaria vector, *Anopheles stephensi*. By 2050, nearly 70% of the world`s population will live in urban areas, a significant increase from the current rate. This systematic review and meta-analysis map the epidemiology of urban malaria in sub-Saharan Africa (SSA). The review included individual participants data from studies conducted in urban settings among all populations to characterize and establish pooled estimates of the prevalence and risk factors, which would help guiding strategies for malaria control and elimination in urban settings.

**Methods:**

An exhaustive literature search was done in PubMed, Medline EBSCO, Google Scholar, Science Direct, and Cochrane Library databases. The Joanna Briggs Institute (JBI) guidelines were followed for evidence selection, data extraction, quality assessment and presentation of findings. Peer-reviewed and gray literature published in English since 2000 focusing on urban malaria Epidemiology in SSA was included in the review. Microsoft Excel 2016 spreadsheet and Stata statistical package were used to extract and analyze data, respectively. Potential sources of heterogeneity and publication bias were assessed. A random effects model was employed for the meta- analysis.

**Results:**

Of the 2,468 records identified from various databases, 39 articles were selected for systematic review and meta-analysis. The overall pooled prevalence of urban malaria in SSA was 23.01% (95% CI: 19.42, 26.59; I^2^ = 99.8%, p < 0.0001). Substantial heterogeneity was observed (I^2^ = 99.8%), indicating considerable variability in population and methods employed. Among two studies included for analysis, a random effect model showed that odds of malaria infection were higher among study participants who had history of travel (POR = 4.17 (95% CI: 2.33, 7.46, I^2^ = 75.5%, p = 0.002). Similarly, five studies were included in the review and showed that owning livestock in the house was associated with malaria infection in urban settings (POR = 4.1, 95% CI: 1.62, 10.39; I^2^ = 75.3%, p = 0.044).

**Conclusion:**

This systematic review and meta-analysis showed a high prevalence of urban malaria infection in SSA with high country-wise heterogeneity. While country-level differences contributed to this variability, other important sources of heterogeneity included variations in population included, method employed and population characteristics. The pooled estimate showed that having a travel history and owning livestock in the house were identified as factors associated with urban malaria infection. Therefore, effective urban malaria control requires an integrated and targeted approach that addresses socio-economic, environmental and behavioural drivers.

**Supplementary Information:**

The online version contains supplementary material available at 10.1186/s12936-025-05508-1.

## Background

Malaria is a life-threatening disease primarily found in tropical countries and continues to have a great impact on people’s health and livelihoods globally, even though it is preventable and treated. The World Health Organization (WHO) World Malaria Report 2024 indicated that an estimated 263 million malaria cases were reported globally—an increase of 11 million cases from the 2023 report. The WHO African Region remained the most affected, accounting for 94% of all cases [1, 2].

By 2050, nearly 70% of the global population will live in cities. The WHO and UN-Habitat (United Nations Human Settlements Programme) warn that while urbanization offers benefits, rapid and unplanned growth can harm health, society, and the environment [3]. The global patterns of disease and mortality, including malaria, will change due to a shift in human population from rural to urban areas [4]. Urbanization has altered health patterns in certain places, making infectious disease risks in urban environments different from those in rural ones [5]. Malaria control in urban environments of Africa is also challenged by the recent invasion and expansion of *Anopheles stephensi* as well as rapid and poorly designed urbanization. *Anopheles stephensi*, an urban malaria vector, was recently introduced to Africa from its native Asia [6]. This vector reproduces in artificial water storage containers, withstands extreme temperatures, and resists multiple insecticides; its uncontrolled spread, combined with rapid urbanization, could drive malaria outbreaks in African cities [7–13]. Several models have predicted the widespread of the disease in urban areas, where it may trigger severe malaria outbreaks among the expanding resident populations [10, 11, 13, 14].

There is little agreement over what constitutes an urban area or how to define the urbanization process. While size and density are used in certain nations, administrative definitions are used in others. Despite problems with the definition of what constitutes urban, most urban settings in SSA are now viewed as presenting an increased risk of various infectious diseases including malaria [4, 5, 15]. Urbanization is generally expected to reduce malaria transmission; however, the disease still persists in African cities, in some cases at higher levels than in nearby rural areas [9]. Urban malaria is the transmission of malaria within urban areas mainly influenced by unplanned urbanization and the emergence of urban mosquito vectors. Evidence from sub-Saharan Africa demonstrated that urban malaria transmission differs significantly from district to district within a populous area, and the focal character of transmission necessitates a careful assessment of the risk in different cities [16].

Although some studies showed that urbanization reduced malaria morbidity and mortality [4, 17–19], more recent research indicates urbanization increases the risk of malaria transmission, particularly following the spread of urban malaria vector species [5]. A study on the impact of urbanization and population density in Africa examined how these factors influence the risk of contracting malaria. The findings revealed a persistent threat of malaria infection in densely populated urban areas across the African continent [20]. Similarly, research from Benin and Ghana highlighted the continued transmission of urban malaria [21, 22].

On the other hands, population mobility or history of travel is the major risk factor for urban malaria transmission. Various studies have indicated that a history of travel significantly increases the odds of malaria morbidity and mortality [5, 23–26]. For malaria control strategies to be effective, urban areas where infections are occurring must be targeted, with success relying on accurate epidemiological data [5].

Over the past two decades (2000 to 2024), numerous studies have examined the prevalence and determinants of urban malaria in sub-Saharan Africa using various approaches. These studies, conducted under different ecological conditions, among diverse populations, and with varying sample sizes, sampling methods, and diagnostic techniques, have led to considerable heterogeneity in malaria positivity rate and risk factors. A systematic review and meta-analysis provide a valuable and efficient approach to obtaining a weighted estimate. Therefore, this study aims to assess the pooled prevalence and risk factors of urban malaria in sub-Saharan Africa since 2000. Especially, it seeks to answer the following specific research questions: 1) what is the current prevalence and incidence of urban malaria in SSA? 2) what are the key risk factors associated with urban malaria transmission? A clear understanding of these factors is crucial for making informed decisions to support both global and regional malaria elimination strategy.

## Methods

This systematic review was conducted using framework recommended by the Joanna Briggs Institute (JBI) for systematic review of prevalence and etiologic studies [27].

### Inclusion criteria

The eligibility criteria were based on the JBI framework. A comprehensive search strategy was designed to identify relevant literature, following established frameworks. It was developed based on the “CoCoPo” mnemonic (Concept–Context-population) for systematic reviews of prevalence studies and the “PEO” mnemonic (Population-Exposure of interest-Outcome) for etiologic studies, as recommended by the Joanna Briggs Institute. The mnemonics aligns with this specific research questions and provides significant information for readers.

This framework was ‘Population (urban residents), Concept (urban malaria), outcome (malaria infection) and Context (sub-Saharan Africa)’. The *Population* encompassed all individuals, regardless of age and sex, living in urban settings. The *Concept* focused on urban malaria, incorporating all related investigations or studies, irrespective of the malaria parasite species. Malaria prevalence or incidence of any species was considered as an outcome in this review. The *Context* considered sub-Saharan Africa and the countries within this region.

### Searching sources and search strategy

To conduct this review, a three-step search strategy was employed to identify both published and unpublished studies. First, relevant literature was gathered from various accessible databases including PubMed, Google Scholar, Cochrane Library, Medline EBSCO, and other sources of gray literature. Second, a comprehensive search strategy was developed using Medical Subject Heading (MeSH), indexed terms, and specific keywords derived from the research question. Third, supplementary searches were conducted, including gray literature searches, citation tracking, and hand-searching. All published and unpublished studies assessing urban malaria were retrieved from multiple databases including websites of various national and international organizations. Only studies published in English since 2000 were included. This search strategy involved combining specific terms across different databases (Table 1). The search strategy and results for PubMed data base is attached as supplementary file (S1).

**Table 1:** search strategy for articles on urban malaria in sub-Saharan Africa

Search strategy item	Search strategy
Databases	PubMed, Medline EBSCO, Google scholar, Science direct, Cochrane library
Language filter	English
Time filter	2000–2025
Spatial filter, sub-Saharan Africa	“Africa south of the Sahara” Or “sub-Saharan Africa” OR “Angola “OR “Benin” OR “Botswana” OR “BURKINA FASO” OR “CABO VERDE” OR “Cameroon” OR “CENTRAL AFRICAN REPUBLIC” OR “Chad” OR “Congo” OR “COTE D'IVOIRE”OR “DEMOCRATIC REPUBLIC OF THE CONGO”OR “Djibouti” OR “EQUATORIAL GUINEA”OR “Eritrea” OR “Eswatini” OR “Ethiopia” OR “Gabon” OR “Gambia” OR “Ghana” OR “Guinea” OR “Kenya” OR “Lesotho” OR “Liberia” OR “Malawi” “Mali” OR “Mauritania” OR “Mozambique” OR “Namibia” OR “Niger” “Nigeria” OR “Rwanda” OR “SAO TOME AND PRINCIPE” OR “Senegal” OR “SIERRA LEONE” OR “Somalia” OR “SOUTH AFRICA” OR “SOUTH SUDAN” OR “Sudan” OR “Tanzania” OR “Togo” OR “Uganda” OR “Zambia” OR “Zimbabwe”
Keywords	1. “Malaria” OR “malaria, vivax”OR “malaria, cerebral”OR “malaria, falciparum” OR “malaria, avian” OR “Acute malaria” OR “Plasmodium” OR “Plasmodium ovale” OR “Plasmodium vivax” OR “Plasmodium malariae” OR “Plasmodium falciparum” OR “urban malaria” 2. “Urban Population” OR “Urbanization” OR “Urban Renewal” OR “Urban Health” OR “town” OR “City” OR “cities” 3. Prevalence” OR “Epidemiology” OR “Risk Factors” OR “Epidemiologic Factors” OR “Travel” OR “Travel-Related Illness” OR “population dynamics” OR “Incidence” OR “Risk”
Inclusion criteria	The paper should be: • A peer‑reviewed or grey literature • A published from 2000 and later • Conducted in sub‑Saharan African countries • Published in the English language • Conducted on urban population or comparison of urban and rural • On malaria prevalence and risk factors/determinants
Exclusion criteria	• The paper should meet the following criteria: • Focus outside urban residents/didn`t include urban population • Be conducted in countries outside sub-Saharan Africa • Have been published online before the year 2000 • Include case reports, case studies, reports, meeting minutes, commentaries, letters to the editor, systematic review, scoping review • Fall outside the specified variables of interest

### Study selection

The search results of articles from various databases were exported to EndNote version 21 reference manager software (Clarivate Analytics, PS, USA), where duplicates records were removed. Two independent reviewers (HM & TD) then screened the remaining articles in two stages: first by title and abstract, and then by full-text review. Abstracts were excluded from the initial screening if they did not report the prevalence of urban malaria or its determinants. Studies that met the inclusion criteria were assessed for methodological quality and only those articles with high quality standards were included in the meta-analysis. Moreover, the papers were also searched outside the search strategy using cross-referencing and contacting experts in the area. Furthermore, additional relevant articles were identified outside the primary search strategy through cross-referencing and consultation with experts in the field. Any disagreements during the review were solved through discussion or with additional reviewer (DY). This systematic review considered observational studies mainly prospective and retrospective cohort, case–control and cross-sectional studies. It did not consider case series, individual case reports and correlational studies. Moreover, qualitative studies, literature review, systematic reviews, text and opinion papers were not considered.

### Quality assessment and critical appraisal

For quality assessment, Joanna Briggs Institute's study-specific critical appraisal tool was used [28]. The tool consists of 9 criteria presented as questions, which can be answered with “yes”, “No”, “Not reported”, or “Not appropriate”. Based on the proportion of ‘yes’ response to the appraisal questions, the studies were categorized into three groups: low, moderate, and high risk of bias. The maximum score a paper could attain was 9 or 100%. The final score was divided by 9 to get the quality index of each paper. A study with 80% or more “yes” responses to the relevant questions, indicating good methodological quality, was classified as having a “low” risk of bias. Studies with 60% to 79% “yes” responses, suggesting some methodological limitations, were classified as having a “moderate” risk of bias. Lastly, studies with fewer than 60% “yes” responses, raising significant concerns about their quality, were deemed as having a “high” risk of bias. Papers with a rating above 50% were included in the final analysis. The critical appraisal results for the screened studies in this research are listed in Table 2.

**Table 2:** Critical appraisal results of screened studies for prevalence of urban malaria in sub-Saharan Africa

Author	Q1	Q 2	Q 3	Q 4	Q 5	Q 6	Q 7	Q8	Q 9	Overall appraisal	Risk of bias
Alemu et al., 2011 [29]	Y	Y	Y	Y	Y	Y	Y	Y	Y	100%	Low
Awosolu et al., 2021[41]	Y	Y	Y	Y	Y	Y	Y	Y	Y	100%	Low
Baragatti et al., 2009[53]	Y	Y	Y	Y	Y	Y	Y	Y	NR	89%	Low
Brenyah et al., 2013[54]	Y	Y	Y	Y	Y	Y	Y	Y	Y	100%	Low
Diallo et al., 2012[55]	Y	Y	NR	Y	Y	Y	Y	Y	NR	78%	Moderate
Enato et al., 2007 [42]	Y	N	NR	Y	Y	Y	Y	Y	NR	67%	Moderate
Enoch and Gloria, 2018[43]	N	N	NR	Y	Y	Y	Y	Y	Y	67%	Moderate
File and Dinka, 2020 [30]	N	N	Y	Y	Y	Y	Y	Y	NA	67%	Moderate
File et al., 2019 [31]	N	N	Y	Y	Y	Y	Y	Y	NA	67%	Moderate
Govoetchan et al., 2022[50]	Y	NA	NR	Y	Y	Y	Y	Y	Y	78%	Moderate
Hassen and Dinka, 2020 [32]	Y	NA	NR	Y	Y	Y	Y	Y	NA	67%	Moderate
Hassen and Dinka, 2022 [33]	Y	Y	Y	Y	Y	Y	Y	Y	Y	100%	Low
Hodson et al., 2022 [36]	Y	N	NR	Y	Y	Y	Y	Y	NA	67%	Moderate
Issifou et al., 2007 [44]	N	NR	NR	Y	Y	Y	Y	Y	NR	67%	Moderate
Kimbi et al., 2013 [37]	Y	Y	Y	Y	Y	Y	Y	Y	Y	100%	Low
Kouna et al., 2024 [45]	Y	N	N	Y	Y	Y	Y	Y	Y	78%	Moderate
Maghendji-Nzondo et al., 2016 [46]	Y	N	N	Y	Y	Y	Y	Y	Y	78%	Moderate
Matthys et al., 2006 [65]	Y	N	N	Y	Y	Y	Y	Y	Y	67%	Moderate
Matthys et al., 2006 [47]	N	N	N	Y	Y	Y	Y	Y	Y	67%	Moderate
Mbah et al., 2022 [38]	Y	NR	Y	Y	Y	Y	Y	Y	Y	87%	Moderate
Mboera et al., 2006 [66]	Y	NR	Y	Y	Y	Y	Y	Y	Y	87%	Moderate
Molla et al., 2024 [67]	Y	Y	Y	Y	Y	Y	Y	Y	Y	100%	Low
Mulachew et al., 2025 [35]	Y	Y	Y	Y	Y	Y	Y	Y	Y	100%	Low
Mwalimu, 2019 [57]	Y	Y	Y	Y	Y	Y	Y	Y	Y	100%	Low
Nakanjako et al., 2011 [58]	Y	Y	N	Y	Y	Y	Y	Y	Y	89%	Low
Njuguna et al., 2016 [48]	Y	NR	NR	Y	Y	Y	Y	Y	Y	78%	Moderate
Nundu et al., 2021 [51]	Y	Y	Y	Y	Y	Y	Y	Y	Y	100%	Low
Nyasa et al., 2023 [39]	Y	Y	NR	Y	Y	Y	Y	Y	Y	89%	Low
Opoku Afriyie et al., 2024 [49]	Y	Y	Y	Y	Y	Y	Y	Y	Y	100%	Low
Othnigue et al., 2006 [52]	Y	N	N	Y	Y	Y	Y	Y	Y	78%	Moderate
Roman et al., 2018 [40]	N	N	N	Y	Y	N	Y	Y	Y	56%	High
Soma et al., 2018 [68]	Y	N	N	Y	Y	Y	Y	Y	NR	67%	Moderate
Thwing et al., 2009[59]	Y	N	N	Y	Y	Y	Y	Y	Y	78%	Moderate
Zhou et al., 2016 [26]	Y	Y	Y	Y	Y	Y	Y	Y	NA	89%	Low

Key: Y = Yes; N = No; NR = Not reported, NA = Not appropriate

Question codes: Q1. Was the sample frame appropriate to address the target population? Q 2. Were the study participants sampled in appropriate way? Q 3. Was the sample size adequate? Q 4. Were the study subjects and setting described in detail? Q 5. Was the data analysis conducted with sufficient coverage of the identified sample? Q 6. Were valid methods used for the identification of the condition? Q 7. Was the condition measured standard, reliable way for all participants? Q 8. Was there an appropriate statistical analysis? Q 9. Was the response rate adequate?

### Data extraction and analysis

Data were extracted using a Microsoft Excel 2016 spreadsheet (Table 3), which included following variables: first author’s last name, year of publication, sample size, study country, study population, diagnostic method, sampling technique, number of malaria positive cases, and odds ratio (OR) with 95% confidence interval (CI).

**Table 3:** Characteristics of included studies

Author and year of publication	Country	Diagnostic Method	setting	Study design	Sampling technique	Study population	Symptomatic status	Sample size	Positive	Prevalence	Species	
											P.f	P.v
Alemu et al., 2011[29]	Ethiopia	Microscopy	Community/HH	Cross sectional	Simple random sampling	Household members	Asymptomatic	804	41	5.2	11	30
Awosolu et al., 2021 [41]	Nigeria	Microscopy	Health Facility	Cross sectional	Simple random sampling	Febrile patients	Symptomatic	300	165	55	–	–
Baragatti et al., 2009 [53]	Burkina Faso	Microscopy	Community	Cross sectional	Stratified sampling	Household members	Asymptomatic	3354	258	7.7	43	–
Baragatti et al., 2009 [54]	Ghana	Microscopy	Community/HH	Cross sectional	Systematic sampling	Household members	Asymptomatic	400	35	8.75	–	–
Diallo et al., 2012 [55]	Senegal	Microscopy	Community	Cross sectional	–	Women	Asymptomatic	2427	50	2.08	–	–
Enato et al., 2007 [42]	Nigeria	Microscopy	Health Facility	Cross sectional	–	Pregnant women	Asymptomatic	199	60	30	–	–
Enoch and Gloria, 2018 [43]	Nigeria	Microscopy	Health Facility	Cross sectional	–	Pregnant women	Asymptomatic	200	55	27.5	–	–
File and Dinka, 2020 [30]	Ethiopia	Microscopy	Health Facility	Cross sectional	–	Febrile Patients	Symptomatic	2590	97	3.7	56	35
File et al., 2019 [31]	Ethiopia	Microscopy	Health Facility	Cross-sectional	–	Febrile Patients	Symptomatic	–	6862	–	2179	2986
Govoetchan et al., 2014 [56]	Benin	RDT	School based	Cross-sectional	Simple random sampling	School children	Asymptomatic	200	15	7.5	–	–
Govoetchan et al., 2022 [50]	Benin	Microscopy	Health Facility	Cross-sectional	–	Febrile patients	Symptomatic	12,975	5079	39	–	–
Hassen and Dinka, 2020 [32]	Ethiopia	Microscopy	Health Facility	Cross-sectional	–	Febrile patients	Symptomatic	175,423	21,797	12.4	10,791	11,006
Hassen and Dinka, 2022 [33]	Ethiopia	Microscopy	Health Facility	Cross-sectional	Simple random sampling	Febrile patients	Symptomatic	356	61	17.13	28	31
Hodson et al., 2022 [36]	Cameroon	PCR	Health Facility	Cross sectional	Convenient sampling	Febrile patients	Symptomatic	503	187	37.2	–	–
Issifou et al., 2007 [44]	Gabon	Microscopy	Health Facility	Cross sectional	–	Children	Asymptomatic	9338	3642	39		
Kazembe and Mathanga, 2016 [62]	Malawi	Microscopy	Mixed community and facility	Case control	Systematic sampling	Children < 5 years	Asymptomatic	767	258	–	–	–
Kimbi et al., 2013 [37]	Cameroon	Microscopy	School based	Cross sectional	–	Children 4–15 years	Asymptomatic	129	33	25.6	–	–
Kouna et al., 2024 [45]	Gabon	Microscopy	Health Facility & school based	Cross sectional	–	Febrile and afebrile children	Both asymptomatic and symptomatic	880	190	21.6	50	–
Maghendji-Nzondo et al., 2016 [46]	Gabon	Microscopy	Health facility	Cross sectional	–	Children 6 months- 15 years	Asymptomatic	280	59	21.2	–	–
Mathanga et al., 2016 [23]	Malawi	PCR	Health facility	Case control	–	Children 6 moths −5 years	Asymptomatic	473	187	–	–	–
Matthys et al., 2006 [65]	CÔTE D’IVOIRE	Microscopy	House hold	Cross sectional	–	All household members	Asymptomatic	672	216	32.1	184	–
Mawili-Mboumba et al., 2017 [47]	Gabon	PCR	Health facility	Cross sectional	–	Febrile patients	Symptomatic	106	66	62.2	–	–
Mbah et al., 2022 [38]	Cameroon	RDT	Community based/HH	Comparative cross sectional	–	All households	Asymptomatic	119	5	4.2	5	–
Mboera et al., 2006 [66]	Tanzania	Microscopy	School based	cross sectional	–	School children 6–15 years	Asymptomatic	787	106	13.5	105	–
Merga et al., 2024 [34]	Ethiopia	PCR	Health facility	Case control	Systematic sampling	Febrile patients	Symptomatic	396	132	–	90	34
Molla et al., 2024 [67]	Ethiopia	PCR	Community based/HH	Cross section	-	Household members	Asymptomatic	504	149	29.6	–	–
Mulachew et al., 2025 [35]	Ethiopia	Microscopy	Community based/HH	Cross sectional	Multistage sampling	Household members	Asymptomatic	422	20	4.7	6	12
Mwalimu, 2019 [57]	Tanzania	RDT	Community based/HH	Cross sectional	Multistage sampling	Household members	Asymptomatic	830	37	4.5	–	–
Nakanjako et al., 2011 [58]	Uganda	RDT	Health facility	Cross sectional	Systematic random sampling	ART patients	Asymptomatic	128	5	4	–	–
Njuguna et al., 2016 [48]	Kenya	Microscopy	Health facility	Cross sectional	–	Febrile patients	Symptomatic	11,480	255	22	–	–
Nundu et al., 2021 [51]	Democratic Republic of Congo	PCR	School based	Cross sectional	Two-stage stratified cluster sampling	School children 6–14 years	Asymptomatic	313	192	61.3	141	–
Nyasa et al., 2023 [39]	Cameroon	Microscopy	Community based/HH	Cross sectional	Systematic sampling	Household members	Asymptomatic	250	117	46.8		
Opoku Afriyie et al., 2024 [49]	Ghana	RDT	Health facility	Cross sectional	–	Febrile patients	Symptomatic	1739	432	24.8		
Othnigue et al., 2006 [52]	Chad	Microscopy	Health facility	Cross sectional	–	Febrile patients	Symptomatic	1658	780	47	234	–
Roman et al., 2018 [40]	Cameroon	Microscopy	Health facility	Cross sectional	Simple random sampling	Children	Asymptomatic	233	92	39.5	–	–
Siri et al., 2010 [69]	Kenya	Microscopy	Health facility	Case control	–	Children < 7 years	Asymptomatic	826	80	–	–	–
Soma et al., 2018 [68]	Burkina Faso	Microscopy	Community based/HH	Cross sectional	–	Household	Asymptomatic	1728	206	12	205	–
Thwing et al., 2009 [59]	Angola	Microscopy	Health facility	Cross sectional	–	Sequential febrile patients	Symptomatic	864	31	3.6		
Zhou et al., 2016 [26]	Ethiopia	Microscopy	Health facility	Cross sectional	–	Febrile patients	Symptomatic	1434	428	29.8	97	327

HH: Household

After independently extracting the data, two reviewers (HM and TD) cross-checked it for inconsistencies and resolved any discrepancies through discussion. The meta-analysis was performed using Stata statistical software version 16 (Stata Corp., College Station, TX, USA). During the analysis, potential sources of heterogeneity were explored through sub-group analysis as well as meta regression, and publication bias was assessed using a funnel plot. The Cochrane Q test and Inconsistency index (I^2^ statistics) were used to evaluate the heterogeneity among the studies. Heterogeneity was observed for the outcome; therefore, a random-effects model was used to determine the pooled prevalence of urban malaria infection. A Cochrane Q value with p < 0.05 indicated significant heterogeneity. Heterogeneity levels were categorized as low, moderate, and high based on I^2^ values of less than 25%, 25–75%, and greater than 75%, respectively. Forest plots were used to display point estimates and confidence intervals. Publication bias was evaluated visually through funnel plots (subjectively) and statistically using Egger’s and Begg’s tests (objectively). A p-value > 0.05 indicates no evidence of publication bias. Trim-and-fill analysis was conducted to estimate the number of studies potentially missing due to publication bias, impute those studies, and derive the overall effect size using both observed and imputed data.

## Results

### Selection and characteristics of included studies

**Fig. 1:**
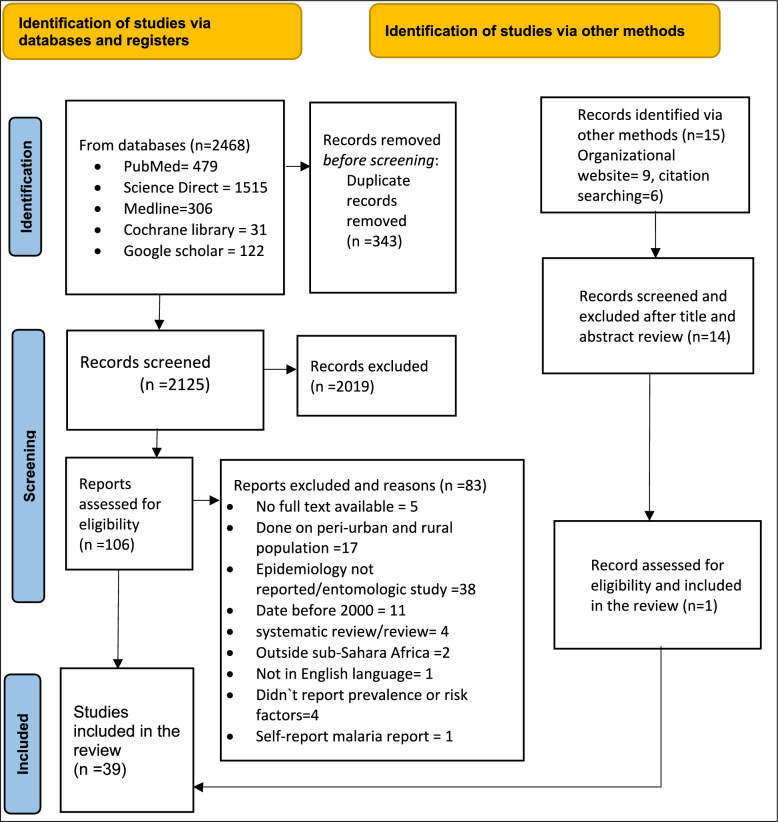
PRISMA flow chart diagram for prevalence of Urban malaria in sub-Saharan Africa

A total of 2468 of published and unpublished articles related to the title were accessed in this initial literature search. After removing 343 duplicate articles, 2125 articles were screened for title and abstract relevance. For full text review, 106 articles were eligible. Finally, after a comprehensive reading of relevant sections of those articles, 39 articles met the inclusion criteria and were included in the systematic review and meta-analysis as shown on PRISMA below (Fig. 1).

This systematic review of the prevalence studies included a total of 233,425 study participants with 34,946 confirmed cases. The majority of the studies were cross-sectional, used for estimating pooled prevalence, while both cross-sectional and case–control studies were utilized to examine determinants. Studies from 16 countries from sub-Saharan Africa were included in the study. Ethiopia contributed the largest proportion of prevalence studies (21.2%), with a sample size of 181,533 and 22,593 confirmed cases, followed by Cameroon (15.15%) with a sample size of 1,234 and 434 confirmed cases. *Plasmodium falciparum* and *Plasmodium vivax* were the predominant malaria species reported in the studies included in this review (Table 3).

### Pooled prevalence of urban malaria

**Fig. 2:**
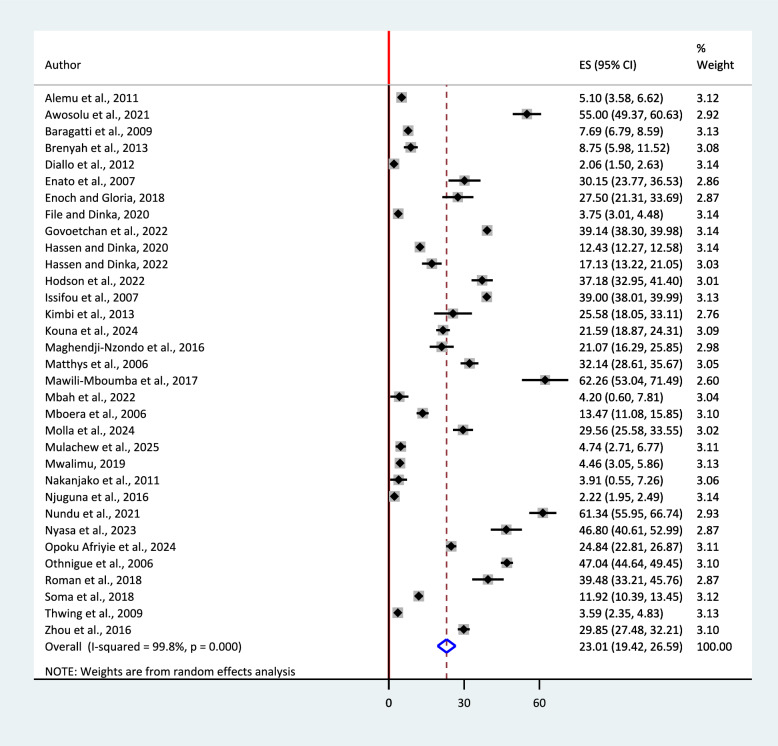
A forest plot showing the pooled prevalence estimate of urban malaria in sub-Saharan Africa, 2025

Substantial heterogeneity was found among included articles (I^2^ = 99.8%, p < 0.0001), and a random-effects model was used to estimate the pooled prevalence of urban malaria in SSA. The overall pooled prevalence was 23.01% (95% CI: 19.42, 26.59) (Fig. 2).

### Sub-group analysis and meta regression analysis

**Fig. 3:**
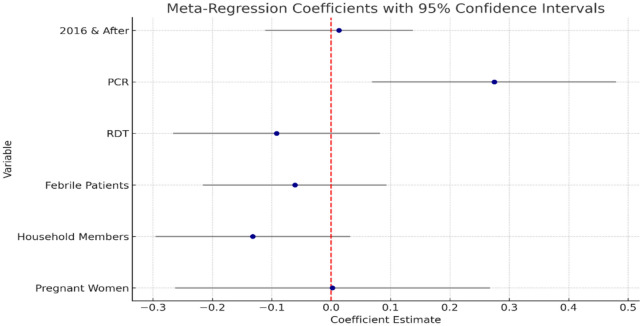
Meta regression analysis

To assess heterogeneity among the included studies, sub-group analysis and meta regression was conducted by diagnostic methods (RDT (Rapid Diagnostic Test), Microscopy and PCR (Polymerase Chain Reaction)) employed, study settings, year of publication and study population. The meta-regression analysis revealed that the type of diagnostic method significantly influences the estimated malaria prevalence, with studies using PCR reporting higher prevalence rates (effect size = 0.275, p = 0.009; 95% CI: 0.1, 0.5). In contrast, other factors—such as study year, use of RDT, and study population type—were not significantly associated with malaria prevalence (p > 0.05), indicating they had no substantial impact on the effect size in this analysis (Fig. 3).

**Fig. 4:**
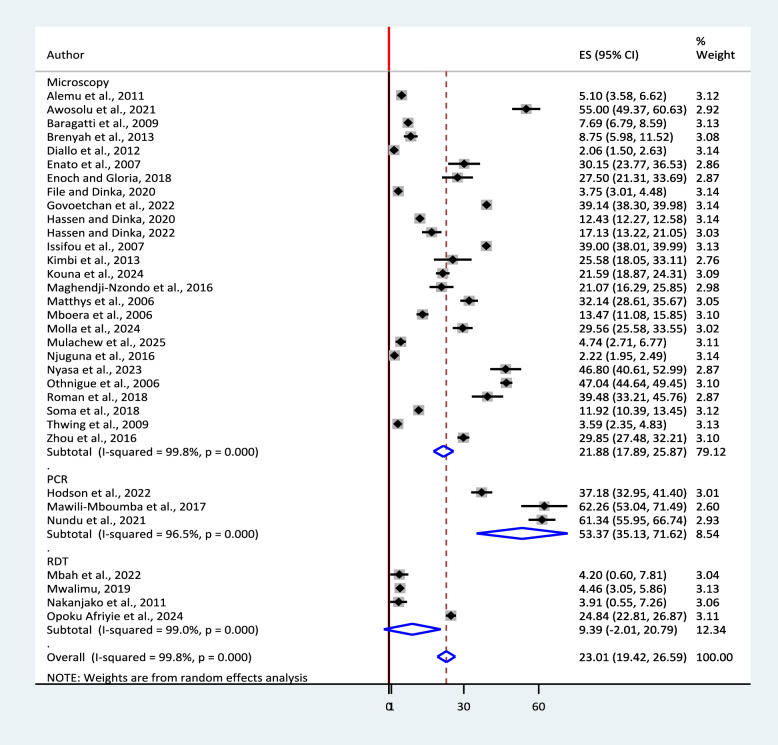
Sub-group analysis by diagnostic methods for the pooled prevalence of urban malaria in SSA

Accordingly, the highest pooled prevalence was reported among those tested with PCR (53.37%, 95% CI: 35.13, 71.62%, I^2^ = 96.5%, p < 0.0001) followed by Microscopy (21.88%, 95% CI: 17.89, 25.87%, I^2^ = 99.8%, p < 0.00010) and RDT (9.39.88%, 95% CI: − 2.01, 20.79, I^2^ = 99%, p < 0.0001). Heterogeneity is high among those sub-groups (Fig. 4).

**Fig. 5:**
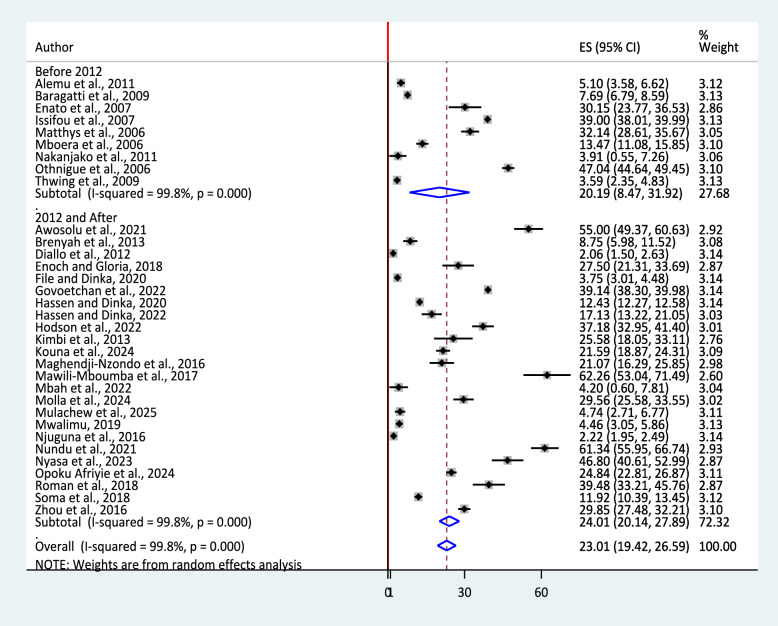
Sub-group analysis by year of publication for the pooled prevalence of urban malaria in sub-Saharan Africa, 2025

Similarly, subgroup analysis by publication year (since the detection of *An. stephensi* in Africa) (before 2012 and 2012 & onwards) was conducted. Even though the heterogeneity was high and similar in both categories, the pooled prevalence of urban malaria in 2012 and onwards was higher (24.01%; 95% CI: 20.14, 27.89.13, I^2^ = 99.8%, p < 0.0001) than before 2012 (20.19%, 95%CI: 8.47, 31.92, I^2^ = 99.8%, p < 0.0001) (Fig. 5).

**Fig. 6:**
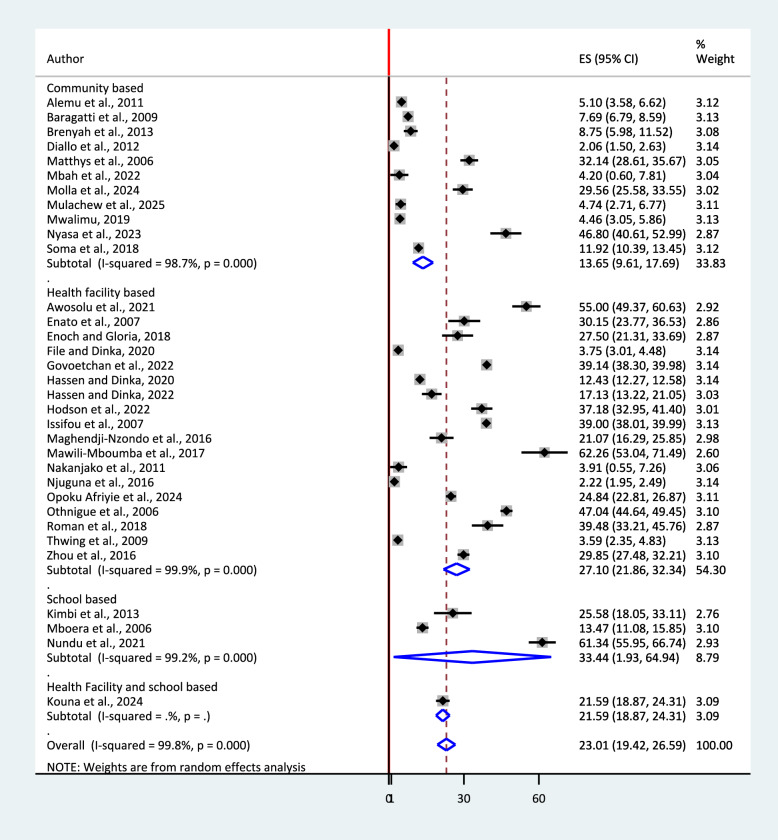
Sub-group analysis by study setting for the pooled prevalence of urban malaria in sub-Saharan Africa, 2025

In this meta-analysis, subgroup analysis was also conducted using study settings. The pooled prevalence of studies conducted in school (among school children) 33.4% (95% CI: 1.93, 64.94) was higher than health facility 27.10% (95% CI: 21.86, 32.34) and community based 13.65% (95%CI: 9.61, 17.69) pooled prevalences (Fig. 6).

**Fig. 7:**
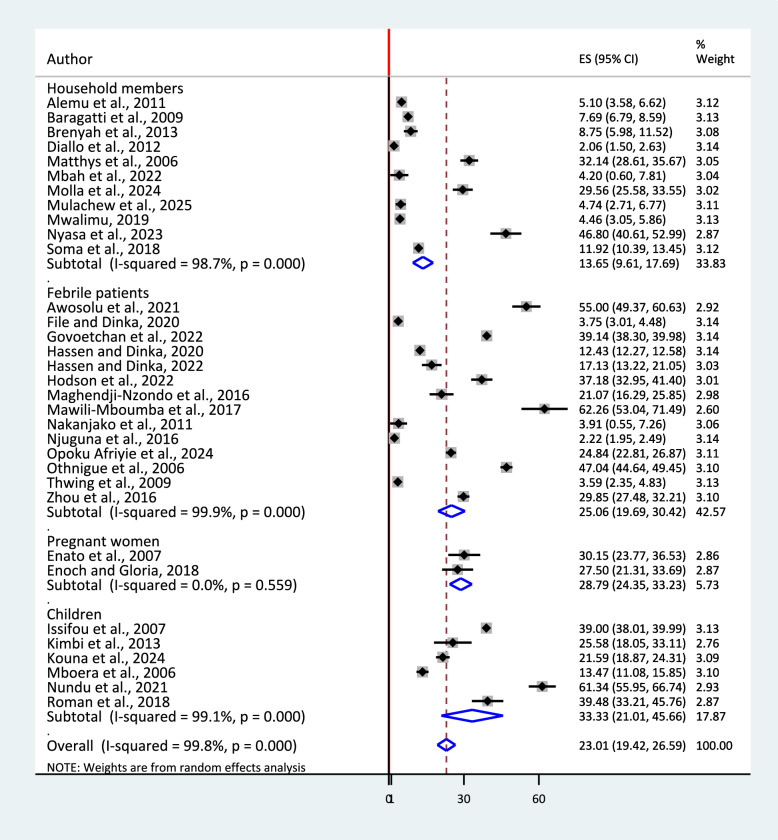
Sub-group analysis by study population for the pooled prevalence of urban malaria in sub-Saharan Africa, 2025

The sub-group analysis by study population indicated that the pooled prevalence among children was 33.33% (95% CI: 21.01, 45.66), among pregnant mothers 28.79% (95% CI: 24.35, 33.23), among households 25.06% (95% CI: 19.69, 30.42) and among febrile patients 13.65% (95% CI: 9.61, 17.69) (Fig. 7).

**Fig. 8:**
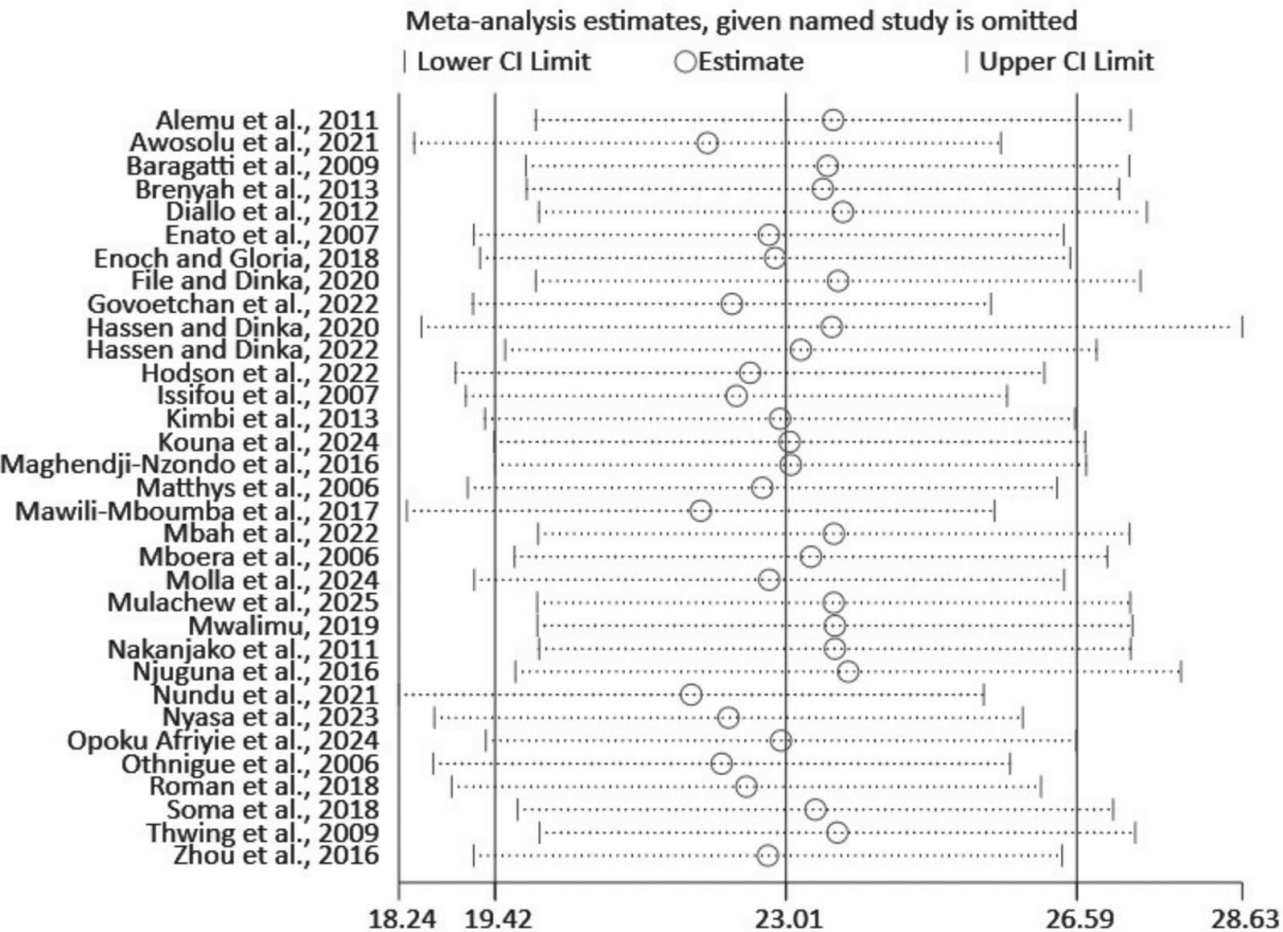
Sensitivity analysis graph to examine the effect of each study on the pooled prevalence of urban malaria in sub-Saharan Africa, 2025

A sensitivity analysis was performed to assess the effect of each study on the malaria prevalence pooled estimate. It is revealed that the size remained consistent around and none of the studies seems to be an extreme outlier that shifts the overall pooled estimate significantly (Fig. 8).

### Assessment of publication bias

**Fig. 9:**
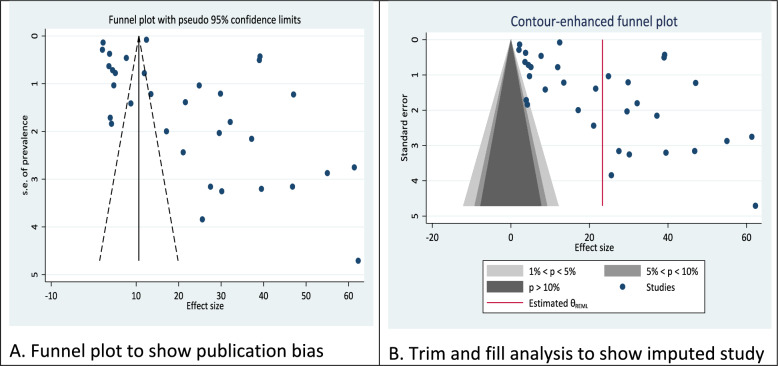
Funnel plot and trim and fill analysis for assessment of publication bias

To generate a funnel plot, the effect of the study was plotted against the standard error (Fig. 8A). The visual evaluation of the funnel plot in this picture showed an asymmetrical picture. However, due to its subjectivity in evaluating the presence of publication bias, Egger’s test and Begg’s test were performed and the results showed absence of statistical evidence of publication bias (Egger’s test, coefficient = 7.71, 95% CI: − 1.49 to 16.91, p = 0.098 and Begg’s test statistic of z = 2.01 and p = 0.076) (Fig. 9). Trim-and-fill analysis suggested that there is no study imputed to account for potential publication bias. There is no indication that studies with different results are missing from the analysis (Fig. 9B).

### Factors associated with urban malaria infection in sub-Saharan Africa

In this review, several variables from primary studies were reviewed to see their association with malaria infection. Thus, four variables that predicted urban malaria infection (travel history, stagnant water, ITN utilization and owning Livestock) were reviewed. However, only two of them (travel history and owning livestock) found statistically significant for malaria infection (Table 4).

**Table 4:** Factors associated with urban malaria in sub-Saharan Africa

Risk factors	Number of studies	Pooled estimate (OR with 95% CI)	Heterogeneity	
			I^2^	p-value
Travel history	5	POR = 4.17 (2.33, 7.46	75.5%,	0.002
ITN utilization	4	POR = 0.61 (0.13, 2.97)	90.8%	< 0.001
Own livestock	2	POR = 4.1 (1.62, 10.39)	75.3%	0.044
Stagnant water	5	POR = 1.4 (0.51, 3.89)	93%	< 0.001

**POR: Pooled Odds Ratio

**Fig. 10:**
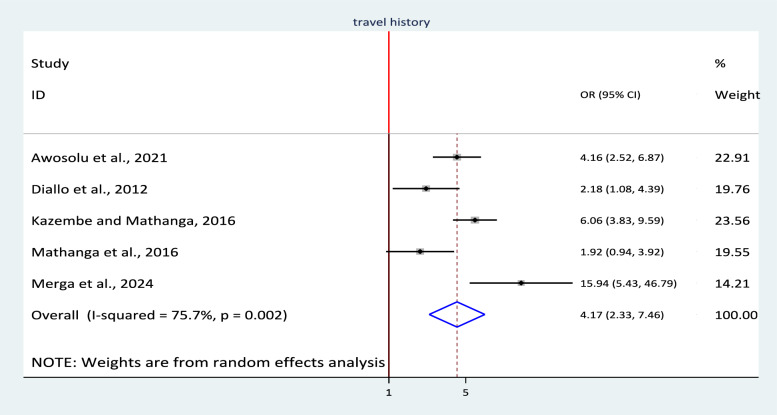
Association between travel history and malaria infection in sub-Saharan Africa

To assess the association between malaria infection and travel history, five studies were included in the review. A random effect model was used to estimate their pooled association with odds ratio (I^2^ = 75.5%, p = 0.002). This meta-analysis showed that there was statistically significant association between malaria infection and travel history. Accordingly, the odds of malaria infection were higher among study participants who had history of travel (OR = 4.17 (95% CI: 2.33, 7.46, p < 0.001) (Fig. 10).

**Fig. 11:**
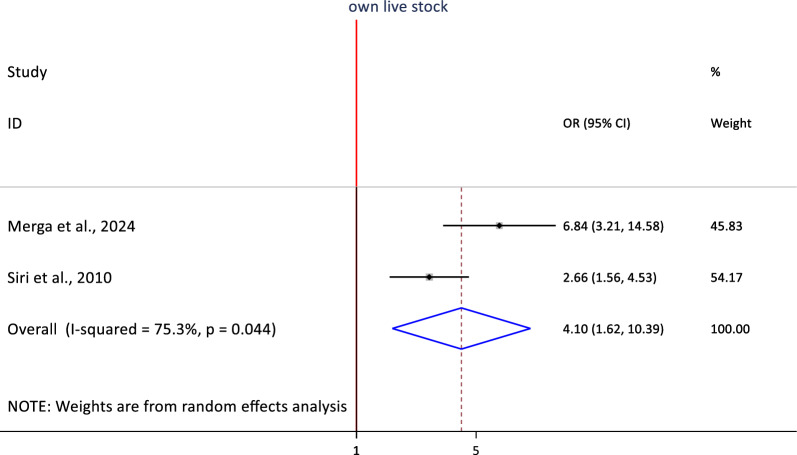
Association between owning livestock and malaria infection in sub-Saharan Africa

Similarly, owning livestock was associated with urban malaria infection. The pooled estimate showed that those who had any livestock in their house were 4.1 times more likely infected with malaria (OR = 4.1, 95%CI: 1.62, 10.39, I^2^ = 75.3%, p = 0.044) (Fig. 11).

## Discussion

This systematic review and meta-analysis focused on the epidemiology of urban malaria in sub-Saharan Africa. The review presents the pooled estimates of urban malaria and its determinants in this region in the past two decades between 2000 and 2024. Sixteen studies were included in the review and majority of them were from Ethiopia [26, 29–35], Cameroon [36–40], Nigeria [37, 41–43] and Gabon [44–47].

The overall pooled prevalence of urban malaria in this review was 23.01% which is in line with the findings from two studies from Gabon 21.6% & 21.2% [45, 46] Ghana 24.8% [48] and Kenya 22% [49]. However, it is very low compared to findings from Nigeria (55% [41], Benin 39% [50], Gabon 39% [44], Gabon 62.2% [47], DRC 61.3% [51], Cameroon 46.8% [39] and Chad 47% [52]. The findings from Ethiopia (5.2% [29], 3.7% [30] & 4.7% [35]), Burkina Faso 7.7% [53], Ghana 8.75% [54], Senegal 2.1% [55], Benin 7.5% [56], Cameroon 4.2% [38], Tanzania 4.5% [57], Uganda 4% [58] and Angola 3.6% [59] are extremely low compared to this review`s pooled estimate result. The inconsistencies in these results might be attributed to the nature of the studies included. While the present study is a systematic review that provides pooled estimates, the studies being compared are individual cross-sectional studies with smaller sample sizes. Additionally, variations in the study population, seasonality, and the diagnostic methods used to detect malaria may have contributed to these discrepancies. Consistent with findings from individual observational studies, the subgroup analysis revealed that the pooled prevalence of urban malaria diagnosed by PCR was more than twice that detected by microscopy and nearly five times higher than that identified using RDTs. This suggests that PCR, being a more sensitive diagnostic method, detects a greater number of malaria cases that may be missed by conventional techniques.

The pooled prevalence of malaria was high among children in this review. This might be attributed to their lower immunity, as immunity to malaria develops gradually through repeated exposure. As a result, children are more vulnerable to infection, and the majority of malaria cases and deaths occur within this age group [60].

On the other hand, the pooled prevalence of urban malaria has been higher since 2012, coinciding with the detection of *An. stephensi* in Africa (Djibouti) [61]. This might be attributed to the expansion of this vector in urban environments, particularly in the Horn of Africa, where its presence is likely contributing to increased malaria transmission in urban settings.

In this review, though many variables were reviewed from the studies included in the review, the pooled estimates of travel history and owning livestock were found statistically significant for malaria infection. The odds of malaria infection were higher among study participants who had history of travel which is similar with findings from Ethiopia [34, 35], Senegal [55], Malawi and [62]. The possible reason could be visitors are not immune, lack of information about the area and poor ITN utilization. Furthermore, increased migration between cities or regions may be contributing to the diversity of *Plasmodium* species in urban environments [63]. The association of travel history with malaria infection suggests the need for enhanced travel medicine interventions among urban dwellers who travel to high transmission areas. This might account for pre-travel health advice, availability of malaria prophylaxis, and better surveillance system. The pooled estimate of the factor owning livestock showed that owning livestock in house increased the odds of malaria infection. This might be due to the fact that mosquitoes are preferring to rest in poorly animal sheds [11, 64]. The link between urban agriculture/livestock and malaria risk highlights the necessity of regulating domestic agricultural practices. Policies that promote safe water management in urban farming, proper livestock waste disposal, and community education on mosquito control can help mitigate associated risks while supporting food security.

### Strength and limitations

Various databases and digital libraries were extensively searched for both published and unpublished articles. However, this review is not without limitations; as primary studies included in this review were cross-sectional and case–control in design; it is difficult to conclude the temporal relationship between the malaria infection and its determinants. Moreover, the pooled estimates may be overestimated or underestimated due to differences in the study setting, study populations, and geographical distribution. Furthermore, the comparability of the results when pooled may be impacted by the fact that some studies were carried out during high transmission seasons while others were undertaken during low transmission seasons. The use of results diagnosed with different laboratory diagnostic techniques, could introduce data discrepancies and affect the pooled estimates, is another limitation of this review. The other limitation of this review is the inability to report on specific malaria species, as most of the studies included did not provide comprehensive data on different malaria species. Furthermore, including studies with different populations and designs could make data synthesis more difficult. However, a subgroup analysis was done to reduce these impacts. Additionally, limiting the review to English-language studies can make it less thorough; adding non-English studies could improve. Another limitation of this review is the inability to access some Malaria indicator survey data due to restricted availability of certain databases. The pooled estimate of travel history and livestock ownership is based on a limited number of studies and these findings should be interpreted with caution, and further research is needed.

## Conclusion

This systematic review and meta-analysis revealed a high pooled prevalence of urban malaria in sub-Saharan Africa, with significant heterogeneity across the included studies. Population mobility/travel history and livestock ownership were identified as key determinants of urban malaria, highlighting the challenges posed by population mobility for malaria control and prevention. These findings suggest that strategies effective in rural areas may not be as effective in urban settings. Therefore, a holistic approach that integrates malaria control strategies with multi-sectorial collaboration is essential for achieving malaria elimination in urban areas.

## Supplementary Information

Additional file 1

## Data Availability

Data is provided within the manuscript or supplementary information files.

## Abbreviations

ICMER: International Center of Excellence for Malaria Research
ITN: Insecticide-treated net
JBI: Joanna Briggs Institute
POR: Pooled odds ratio
SSA: Sub-Sahara Africa
WHO: World Health Organization
