# Supplementary material for: Urban malaria and population mobility in sub-Saharan Africa: systematic review and meta-analysis
Source: Malar J. 2025 Aug 18;24:264. doi: 10.1186/s12936-025-05508-1 (PMC12362982; doi:10.1186/s12936-025-05508-1)
Supplement: Supplementary file 1 — Additional file 1 [file 12936_2025_5508_MOESM1_ESM.docx]

Final search results from PubMed data base

|  | Search | Results |
| --- | --- | --- |
|  | ("Malaria"[MeSH Terms] OR "malaria, vivax"[MeSH Terms] OR "malaria, cerebral"[MeSH Terms] OR "malaria, falciparum"[MeSH Terms] OR "malaria, avian"[MeSH Terms] OR "Acute malaria"[Supplementary Concept] OR ("Plasmodium"[MeSH Terms] OR "Plasmodium ovale"[MeSH Terms] OR "Plasmodium vivax"[MeSH Terms] OR "Plasmodium malariae"[MeSH Terms] OR "Plasmodium falciparum"[MeSH Terms]) OR ("Malaria"[Title/Abstract] OR "Plasmodium falciparum"[Title/Abstract] OR "Plasmodium vivax"[Title/Abstract] OR "Plasmodium ovale"[Title/Abstract] OR "Plasmodium malariae"[Title/Abstract]) OR "urban malaria"[Title/Abstract]) AND ("Prevalence"[MeSH Terms] OR "Epidemiology"[MeSH Terms] OR "Epidemiology"[MeSH Subheading] OR "Risk Factors"[MeSH Terms] OR "Epidemiologic Factors"[MeSH Major Topic] OR ("Travel"[MeSH Terms] OR "Travel-Related Illness"[MeSH Terms]) OR ("population dynamics/history"[MeSH Major Topic] OR "population dynamics/statistics and numerical data"[MeSH Major Topic] OR "population dynamics/trends"[MeSH Major Topic]) OR ("epidemilogy"[Title/Abstract] OR "Prevalence"[Title/Abstract] OR "Incidence"[Title/Abstract] OR "risk factor"[Title/Abstract] OR "Risk"[Title/Abstract])) AND ("Urban Health Services"[MeSH Terms] OR "Urban Health"[MeSH Terms] OR "Urban Population"[MeSH Terms] OR "hospitals, urban"[MeSH Terms] OR "Urbanization"[MeSH Terms] OR "Cities"[MeSH Terms] OR ("Urbanization"[Title/Abstract] OR "Cities"[Title/Abstract])) AND ("Africa South of the Sahara"[MeSH Terms] OR ("Africa South of the Sahara"[Title/Abstract] OR "Angola"[Title/Abstract] OR "Benin"[Title/Abstract] OR "Botswana"[Title/Abstract] OR "burkina faso"[Title/Abstract] OR "cabo verde"[Title/Abstract] OR "Cameroon"[Title/Abstract] OR "central african republic"[Title/Abstract] OR "Chad"[Title/Abstract] OR "Congo"[Title/Abstract] OR "cote d ivoire"[Title/Abstract] OR "democratic republic of the congo"[Title/Abstract] OR "Djibouti"[Title/Abstract] OR "equatorial guinea"[Title/Abstract] OR "Eritrea"[Title/Abstract] OR "Eswatini"[Title/Abstract] OR "Ethiopia"[Title/Abstract] OR "Gabon"[Title/Abstract] OR "Gambia"[Title/Abstract] OR "Ghana"[Title/Abstract] OR "Guinea"[Title/Abstract] OR "Guinea-Bissau"[Title/Abstract] OR "Kenya"[Title/Abstract] OR "Lesotho"[Title/Abstract] OR "Liberia"[Title/Abstract] OR "Malawi"[Title/Abstract] OR "Mali"[Title/Abstract] OR "Mauritania"[Title/Abstract] OR "Mozambique"[Title/Abstract] OR "Namibia"[Title/Abstract] OR "Niger"[Title/Abstract] OR "Nigeria"[Title/Abstract] OR "Rwanda"[Title/Abstract] OR ("sao tome"[Title/Abstract] AND "Principe"[Title/Abstract]) OR "Senegal"[Title/Abstract] OR "sierra leone"[Title/Abstract] OR "Somalia"[Title/Abstract] OR "south africa"[Title/Abstract] OR "south sudan"[Title/Abstract] OR "Sudan"[Title/Abstract] OR "Tanzania"[Title/Abstract] OR "Togo"[Title/Abstract] OR "Uganda"[Title/Abstract] OR "Zambia"[Title/Abstract] OR "Zimbabwe"[Title/Abstract])) AND (2000:2025[pdat]) | 479 |
